# Environmentally activatable hydrogel for spatiotemporally programmed nitric oxide delivery for diabetic wound healing

**DOI:** 10.1016/j.mtbio.2026.102916

**Published:** 2026-02-10

**Authors:** Langjie Chai, Yiran Shi, Qianqian Li, Yifan Han, Liangcong Hu, Yifeng Lei, Liang Guo

**Affiliations:** a Department of Plastic Surgery, Zhongnan Hospital of Wuhan University, Wuhan, 430071, China; b Department of Plastic Surgery, The First Affiliated Hospital of Shandong First Medical University & Shandong Provincial Qianfoshan Hospital, Jinan, 250013, China; c School of Power and Mechanical Engineering, Wuhan University, Wuhan, 430072, China; d Wuhan University Shenzhen Research Institute, Shenzhen, 518057, China

**Keywords:** Diabetic wound, Chitosan hydrogel, NO delivery, Inflammation regulation, Wound healing

## Abstract

Nitric oxide (NO) plays a central role in wound healing, by regulating vascular homeostasis, inflammation, and antimicrobial effects. However, chronic diabetic wounds are difficult to heal due to the hyperglycemic microenvironment, which reduces endogenous NO production. Therefore, developing intelligent dressings capable of spatiotemporally programmed NO delivery holds great promise in promoting diabetic wound healing. Herein, we engineered an environmentally activatable hydrogel that enabled on-demand NO release for diabetic wound healing. The CS-SNAP hydrogel was achieved by covalent grafting of NO donor (S-nitroso-N-acetylpenicillamine, SNAP) onto the matrix of carboxymethyl chitosan methacryloyl (CMCSMA), and by subsequent fast photopolymerization during only 10 s. The CS-SNAP hydrogel enabled sustained release of NO over 300 min by simply modulating ambient light and temperature. When applied to diabetic wounds, not only did the CS-SNAP hydrogel exhibit effective antibacterial activity, but it also showed good angiogenic ability and promoted M1-to-M2 polarization of macrophages. Together, this environmentally activatable platform demonstrates great potential to shorten the inflammatory phase of diabetic wounds, prevent bacterial colonization, and accelerates diabetic wound healing.

## Graphical abstract

**Figure:**
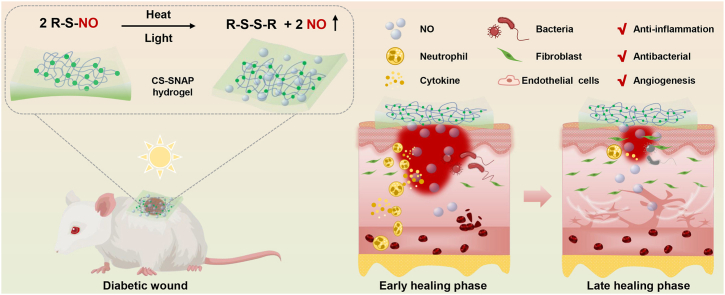

## 1 Introduction

Wound healing is a complex and highly regulated cascade process [1]. When this cascade is disrupted and a wound remains unhealed for more than three months, leading to delayed healing, it is classified as a chronic wound [2,3]. Chronic wounds are a major complication of diabetes [4]. Impaired wound healing in diabetes is primarily due to the reduced regenerative microvascular capacity, a persistent inflammatory response, and a high rate of infection within the diabetic wound microenvironment [5,6]. In severe cases, chronic diabetic wounds can lead to limb amputation, placing a significant burden on both patients and the healthcare system [7]. To accelerate the diabetic wound healing process, advanced wound management strategies are necessary.

Nitric oxide (NO) is an endogenous gaseous molecule involved in various physiological and pathological processes [8,9]. It plays multiple roles in tissue repair, including regulating signal transduction, modulating cytokine secretion and inflammatory cell behavior, controlling cell proliferation and migration, promoting collagen formation, and enhancing vasodilation and angiogenesis [9,10]. NO-based therapies are widely applied in cardiovascular diseases and immunoregulation [11,12]. Additionally, NO is recognized as a potent antimicrobial agent [13,14]. Previous studies have shown that chronic diabetic wounds have a significant deficiency in NO compared to acute wounds [15], leading to persistent chronic inflammation, increased exudate, and bacterial colonization. Researchers have explored the use of NO to promote wound healing in models characterized by immunosuppression and chronic inflammation [[16], [17], [18]]. However, the short half-life and rapid diffusion of NO present significant limitations. Various types of NO donors capable of storing and releasing NO have been developed [[19], [20], [21]], such as organic nitrites, organic nitrates, S-nitrosothiols (RSNOs), diazeniumdiolates (NONOates) [20,22]. S-nitroso-N-acetylpenicillamine (SNAP) was selected as the NO donor in this study due to its superior stability compared to other donors (e.g., NONOates) and its favorable biocompatibility, yielding non-toxic byproducts upon decomposition. Furthermore, SNAP exhibits intrinsic sensitivity to both ambient light and heat, theoretically enabling spatiotemporally programmed NO delivery. Nevertheless, achieving a controlled and sustained release of NO remains a significant challenge.

Currently, commercial products for diabetic wound therapy mainly focus on managing macroscopic factors such as humidity and pressure [[23], [24], [25]]. However, there is still a significant need to address the multiple regulatory axes and signaling cascades involved in chronic diabetic wounds. Hydrogels, widely used in biomedical fields due to their ability to maintain a high water content and a structure similar to the extracellular matrix [[25], [26], [27]], offer promising potential for diabetic wound healing. Hydrogels can protect diabetic wounds, promote cell growth and migration, prevent friction, reduce pressure, and avoid tissue necrosis [28]. Multifunctional hydrogels with antibacterial and controlled drug release features facilitated wound healing, by providing anti-inflammatory, antioxidant and tissue regenerative effects [[29], [30], [31]]. Chitosan, a product derived from chitin [32], has excellent biocompatibility, biodegradability, biosafety, antibacterial activity, and anti-inflammatory properties [33,34]. However, the low solubility of chitosan in water limits its use in a wide range of biomedical applications [35]. Modifying or grafting other molecules or components onto chitosan may be an effective method to improve its water solubility and endow it with biological functions that target the regeneration of skin soft tissue.

**Fig. 1:**
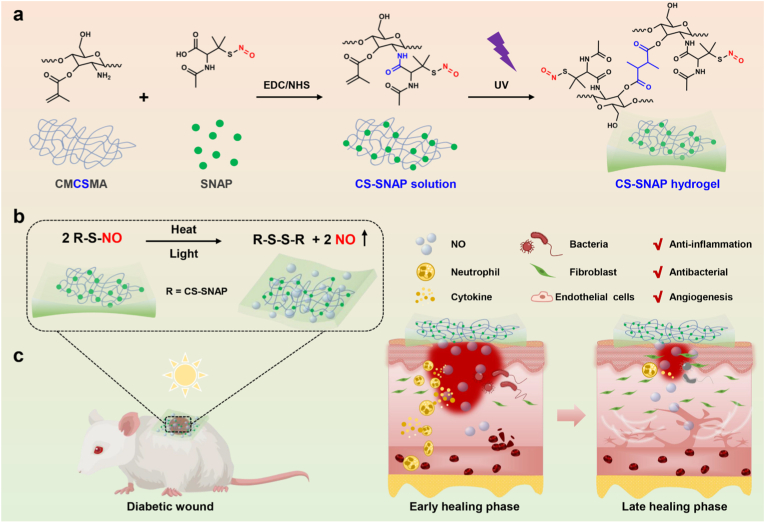
Schematic illustration of CS-SNAP hydrogels for programmed nitric oxide delivery for diabetic wound healing. (a) Preparation process of CS-SNAP hydrogels. (b) Controlled release of NO from CS-SNAP hydrogels. (c) Multiple therapeutic effects of CS-SNAP hydrogels upon application on diabetic wounds.

In this study, we developed a chitosan-based, environmentally activatable hydrogel platform as a NO donor carrier that exhibits sustained NO release to accelerate the diabetic wound healing process. The CS-SNAP hydrogel was achieved by covalent grafting the NO donor SNAP onto CMCSMA, followed by rapid photopolymerization under UV irradiation for only 10 s (Fig. 1a). This rapid photocrosslinking capability allows the hydrogel to conform seamlessly to irregular wound geometries. Specifically, this platform enables spatiotemporally controlled release of NO by leveraging ambient light and temperature (Fig. 1b), maintaining continuous NO delivery for over 300 min for diabetic wound therapy (Fig. 1c). Upon application to diabetic wounds, the positively charged chitosan molecules and the sustained release of NO from the hydrogel exhibit synergistic antibacterial activity, preventing wound infection in the early stages (Fig. 1c). In the subsequent healing stages, the hydrogel promotes fibroblast proliferation and migration, enhances local angiogenesis and collagen deposition, and ultimately accelerates the closure of diabetic wounds. Overall, our strategy provides new insights into the development of functional wound dressings for the management of chronic diabetic wounds.

## 2 Results

### 2.1 Preparation and characterization of CS-SNAP hydrogels

To prepare CS-SNAP hydrogel, first, the carboxyl group on SNAP was activated, and covalently grafted onto the macromolecular structure of carboxymethyl chitosan methacryloyl (CMCSMA) via amide linkage, to form CS-SNAP conjugate (Fig. 1a, left). Subsequently, CS-SNAP solution was fast self-assembled into CS-SNAP hydrogels by photopolymerization under ultraviolet (UV) irradiation (Fig. 1a, right), benefiting from the methacryloyl groups on chitosan methacryloyl structures. Similarly, photopolymerization of CMCSMA solution without loading of SNAP was prepared as the control group, and labeled as CS hydrogels.

**Fig. 2:**
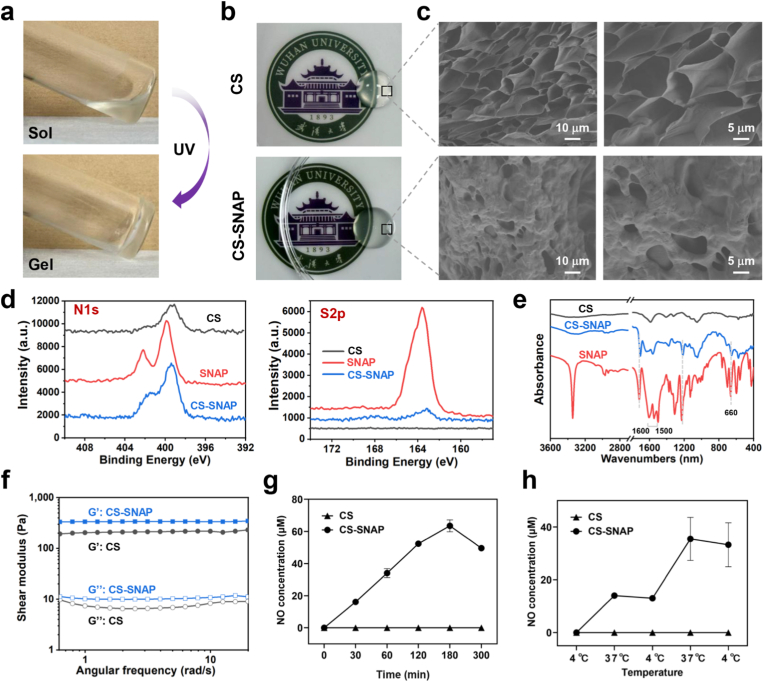
Physicochemical characterization of CS-SNAP hydrogels. (a) Images of photopolymerization process to form CS-SNAP hydrogels. (b) Morphology of photopolymerized CS and CS-SNAP hydrogels. (c) SEM images of CS and CS-SNAP hydrogels. (d) XPS high-resolution spectra during preparation of CS-SNAP hydrogels, including N1s and S2p spectra. (e) FTIR spectra during synthesis of CS-SNAP hydrogels. (f) Rheological property of the prepared hydrogels. (g) NO release curves from CS and CS-SNAP_1.0_ hydrogels as a function of time at 25 °C and in ambient light condition (light-driven release). (h) NO release curves from CS and CS-SNAP_1.0_ hydrogels as a function of temperature in darkness (temperature-driven release).

The CS-SNAP conjugate showed a sol state at room temperature (Fig. 2a), the solution quickly underwent sol-gel transition under UV photopolymerization during only 10 s (Fig. 2a). CS hydrogels showed similar sol-gel transition process as CS-SNAP hydrogels. Specifically, the hydrogels can be photopolymerized to adapt to various wound shapes. Both CS hydrogels and CS-SNAP hydrogels were transparent, and allowed to observe the images below (Fig. 2b). The light transmittance of the prepared hydrogels was also analyzed by UV-Vis spectrophotometer. Both CS and CS-SNAP hydrogels had a good transparency in the wavelength range of 400 ∼ 700 nm (Fig. S1). CS-SNAP hydrogel exhibited slightly lower transmittance compared to CS hydrogels, indicating that the conjugation of SNAP into the CMCSMA network (Fig. 1a) may slightly exhaust the transparency of the hydrogels. The transparency of hydrogels enabled subsequent wound observation during hydrogel dressing application. Scanning electron microscopy (SEM) was used to interpret the microscopic morphology of the hydrogels. After crosslinking by photopolymerization (Fig. 1a), both of CS and CS-SNAP hydrogels exhibited a connected porous structure by SEM observation (Figure S2, Fig. 2c). To provide quantitative insight into the microstructure, the pore size was analyzed using ImageJ software (n = 50). CS hydrogel exhibited an average pore size of 6.4 ± 2.6 μm, while CS-SNAP hydrogel showed a comparable pore size of 4.9 ± 2.5 μm (Fig. S3). Statistical analysis indicated no significant difference between two groups. This confirms that the chemical conjugation of SNAP did not significantly alter the macroscopic 3D network or porosity of the hydrogels, thereby maintaining favorable properties for exudate absorption and gas exchange.

X-ray photoelectron spectroscopy (XPS) was used to determine the chemical composition during hydrogel preparation. CS hydrogel exhibited C, N and O elements as expected, whereas SNAP contained substantial N and S elements due to the -SNO group (Figs. S4–S5, Fig. 2d–Table S1). Compared to CS hydrogels, the obtained CS-SNAP hydrogels showed increased N and S element (Fig. 2d–Table S1), due to the presence of SNAP molecule in the structure. The atomic ratio of N1s increased from 4.04% of CS hydrogel to 8.52% of CS-SNAP hydrogel, whereas the atomic ratio of S2p increased from 0% of CS hydrogel to 1.07% of CS-SNAP hydrogel (Table S1). These results suggested the successful conjugation of SNAP into CS-SNAP hydrogel.

FTIR was further used to evaluate the chemical structure during the formation of CS-SNAP hydrogels. Generally, the FTIR spectrum of CS-SNAP hydrogel was similar to that of CS hydrogel (Fig. 2e), due to the similar polymeric main structure (Fig. 1a). Besides, in CS-SNAP hydrogel, characteristic FTIR bands of SNAP were abundant (Fig. 2e), included the peaks around 1600 cm^−1^ and 1514 cm^−1^ representing the secondary amide C=O stretching and the N=O stretching of SNAP [36], and the peak at 660 cm^−1^ corresponding to the -SNO bending [36,37]. For CS-SNAP hydrogel, the FTIR spectrum was similar to CS hydrogel, but with additional strong characteristic peaks originated from SNAP (Fig. 2e), suggesting the formation of CS-SNAP conjugation.

Rheological test was carried out to validate the hydrogel formation of CS and CS-SNAP hydrogels. The frequency sweep revealed that the both CS and CS-SNAP hydrogels exhibited a higher storage modulus (G′) than loss modulus (G″) (Fig. 2f), indicating the gel-like character of both hydrogels [[38], [39], [40]].

Moreover, NO release from CS-SNAP hydrogels was evaluated in PBS solution using Griess reagent as previously reported [11,17,18]. First, the absorbance of standard sodium nitrite solutions was plotted to generate a standard curve of NO concentration (Fig. S6). To simulate the practical clinical application scenario, first, we evaluated the NO release behavior under ambient conditions (25 °C with ambient light). CS-SNAP hydrogel exhibited a gradual and stable NO release process over 180 min, reaching a peak concentration of 63.5 μM at 180 min (Fig. 2g, Fig. S7a). It is established in previous literature that the decomposition of SNAP at ∼25 °C was negligible in darkness, while NO release at ∼25 °C was predominantly driven by visible light [41]. This characteristic is highly advantageous, as it prevented premature release of NO during storage in darkness, while allowing effective release upon light exposure in practical application. Moreover, a moderate environmental temperature (around 25 °C) is often considered optimal for diabetic wound healing, as it avoids the deleterious effects of heat (which may aggravate inflammation) or cold (which may suppress cellular metabolism). Thus, CS-SNAP hydrogel is well-suited for use in practical clinical environments, taking ambient temperature and light to deliver NO effectively without the need for additional heating or irradiation devices.

Furthermore, to investigate the effect of temperature change on NO release from CS-SNAP hydrogels, measurements were conducted in darkness conditions through alternating cycles at 4 °C and 37 °C, according to previously reported protocols [42]. CS-SNAP hydrogel gradually released NO at 37 °C (Fig. 2h, Fig. S7b). Upon transfer to 4 °C, NO release paused, and NO concentration showed a slight decrease due to the volatilization of NO. Upon returning to 37 °C, NO release recovered (Fig. 2h, Fig. S7b), suggesting warm condition at 37 °C facilitated the release of NO from CS-SNAP hydrogels. Similar trends were observed in CS-SNAP_0.25_ and CS-SNAP_0.5_ hydrogels, and NO release from the CS-SNAP hydrogel increased as the SNAP ratio in CS-SNAP hydrogel increased (Fig. S7).

The temperature-dependent release profile highlights the potential for distinct storage and application phases of CS-SNAP hydrogels. At 4 °C (irrespective of light) or at 25 °C (in darkness), the release of NO gas was negligible, indicating that the hydrogel maintains excellent stability and prevents premature leakage during low-temperature storage. In contrast, upon exposure to ambient light at room temperature (25 °C) or upon reaching physiological temperature (37 °C), the rate of NO release significantly increased. This light or thermal activation mechanism ensures that the therapeutic action is initiated specifically upon contact with the wound bed, without the need for complex external triggers.

Here, it should be noted that the NO release profile was quantified using the Griess assay, which detects nitrite (NO_2_^−^), the primary stable oxidation product of NO in aqueous media, rather than the NO radical itself [11,17,18]. While this method is widely employed for estimating total NO payload, it represents an indirect measurement. It does not capture the real-time flux of gaseous NO and may slightly underestimate the total NO release, since a fraction of NO might escape or oxidize further into nitrate (NO_3_^−^) which is not detected by the standard Griess reagent. Nevertheless, the accumulation of nitrite serves as a reliable correlation for the sustained NO delivery capability of the CS-SNAP hydrogels.

It is also noted that the photopolymerization time (10 s) during hydrogel fabrication (Fig. 1a) is extremely short compared to the half-life of SNAP decomposition. Therefore, the brief UV light exposure during fabrication process caused negligible loss of the NO donor, ensuring that the final hydrogel retains its NO loading capacity.

### 2.2 *In vitro* and *in vivo* biocompatibility

**Fig. 3:**
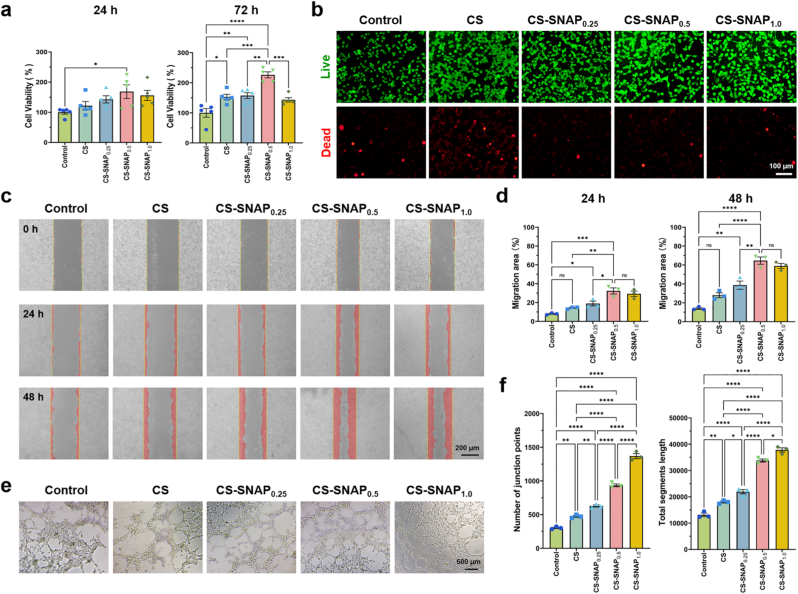
Characterization of biocompatibility of CS-SNAP hydrogels. (a) Viability of HSF cells at 24 h and 72 h with different treatment groups, including control, CS, CS-SNAP_0.25_, CS-SNAP_0.5_, and CS-SNAP_1.0_. (b) Live/dead staining of HSF cells with different treatment for 48 h. Live and dead cells were stained with calcein AM and propidium iodide, resulting in green and red fluorescence, respectively. (c) Optical images during migration assay of HSF cells treated with different groups. (d) Relative migration rate of HSF cells at 24 h and 48 h. (e) Optical images of tube formation assay of HUVEC cells treated with different groups. (f) Quantification of angiogenesis of different groups, including the number of junction points and total segments length. ∗*p* < 0.05, ∗∗*p* < 0.01, ∗∗∗*p* < 0.001, ∗∗∗∗*p* < 0.0001, ns means non-significant (*p* > 0.05).

Biocompatibility is a prerequisite for cell proliferation and migration in wound healing process [43]. First, CCK-8 assay was performed to evaluate the cell viability of CN-SNAP hydrogels. Compared with the control group, HSF cells in CS-SNAP hydrogel groups exhibited a significant proliferation trend over 3 days (Fig. 3a). Notably, among different ratio of SNAP, the CS-SNAP_0.5_ hydrogel group showed highest cell survival rates of 168.4% ± 50.7% on day 1, and 226.2% ± 22.4% on day 3 (Fig. 3a), whereas high concentrations of SNAP did not consistently and effectively promote cell growth, as demonstrated by the CS-SNAP_1.0_ hydrogel group with cell survival rates less than CS-SNAP_0.5_ group (Fig. 3a). Therefore, we mainly selected the CS-SNAP_0.5_ group for subsequent experiments.

Live/dead staining was performed to further verify the cell compatibility of the hydrogels. The spindle-shaped HSF cells were almost entirely stained in green (live cells), with only a few cells were stained in red (dead cells) for CS-SNAP_0.25_, CS-SNAP_0.5_ and CS-SNAP_1.0_ groups, whereas more dead cells existed in control group and in CS hydrogel groups (Fig. 3b). This excellent cell compatibility of CS-SNAP was attributed to the non-toxic property of the chitosan scaffold and sustained NO release from the hydrogels.

Hemocompatibility is also a critical safety concern for biomaterials. Here hemocompatibility of CS-SNAP hydrogel was assessed by evaluating the hemolysis rate using a direct contact method. After centrifugation, the supernatant appearance of CS-SNAP hydrogel groups was similar to that of the saline group (negative control), showing a pale-yellow color, whereas the Triton-X treated group (positive control) showed a bright red color, indicating a high hemolysis rate (Fig. S8a). Quantitative analysis indicated that the hemolysis rates of CS-SNAP hydrogel group was less than 5% (Fig. S8b), demonstrating the excellent hemocompatibility of CS-SNAP hydrogel as a wound dressing.

### 2.3 *In vitro* effects of CS-SNAP hydrogel on cell migration and angiogenesis

The primary challenges in healing diabetic wounds included impaired cell migration, impaired microvascular regeneration and excessive exudate, which together leads to impaired healing [44,45]. Here, we evaluated the effect of CS-SNAP hydrogel extract solution on cell migration using a scratch assay. Increasing SNAP ratio in CS-SNAP hydrogels significantly stimulated the migration of HSF cells (Fig. 3c). Compared to the control group, the CS-SNAP hydrogel groups exhibited markedly higher migration rates at 24 and 48 h (Fig. 3d), demonstrating that the release of NO from CS-SNAP hydrogels promoted the proliferation and migration of HSF cells.

For angiogenesis assay, HUVEC cells were cultured with CS-SNAP hydrogel extract solution [39,40]. After 8 h of incubation, microscopy observations revealed that few tubular structures were formed in control group, whereas the CS-SNAP hydrogel groups showed significant tube formation (Fig. 3e). Specifically, the angiogenesis activity became more significant in CS-SNAP_0.5_ and CS-SNAP_1.0_ hydrogel groups (Fig. 3e). Quantification analysis also revealed that CS-SNAP_0.5_ and CS-SNAP_1.0_ hydrogel groups exhibited the most significant stimulatory effects on tube formation, which exhibited most junction points, and longest total segments length (Fig. 3f). In contrast, the control and CS hydrogel groups showed fewer junction points and total segments length (Fig. 3f). These results proved that CS-SNAP hydrogels exhibited potent angiogenesis activity.

### 2.4 Antibacterial effect

**Fig. 4:**
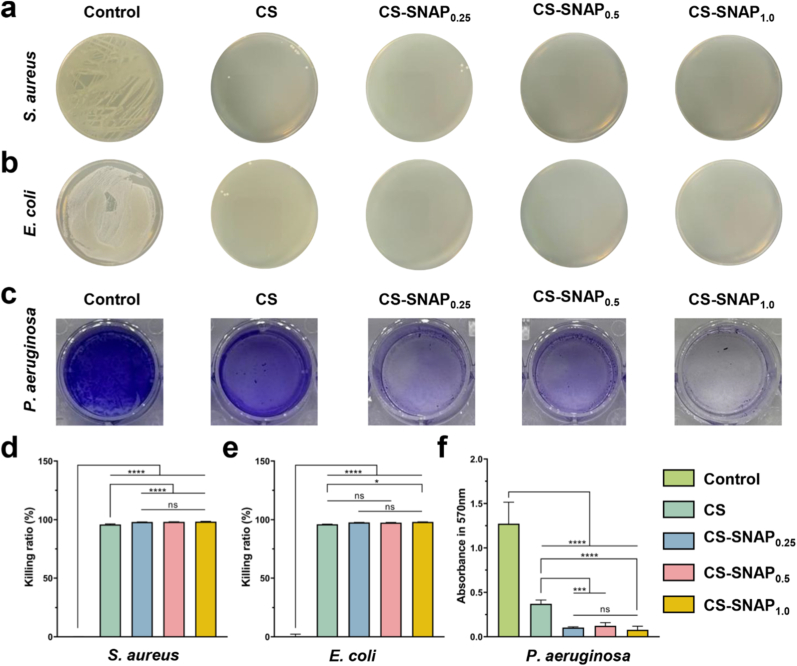
Anti-bacterial activity of CS-SNAP hydrogels. (a) Images of bacterial colonies of *S. aureus* and *E. coli* after cultured with different samples. (c) Images of bacterial biofilms of *P. aeruginosa* after cultured with different groups. (d-e) Killing ratio of hydrogels against *S. aureus* and *E. coli*. (f) Quantification of bacterial biofilms of *P. aeruginosa*. ∗*p* < 0.05, ∗∗∗*p* < 0.001, ∗∗∗∗*p* < 0.0001, ns means non-significant (*p* > 0.05).

High blood glucose levels and hypoxia in diabetic wounds provided favorable conditions for bacterial colonization and biofilm formation [46,47]. Therefore, antibacterial activity of the hydrogels was evaluated by co-culturing them with *Escherichia coli* (*E. coli*) or *Staphylococcus aureus* (*S. aureus*) suspensions at 37 °C in the dark. Compared to the control group, CS hydrogel showed a reduction in bacterial growth on agar plates but still retained countable colonies (Fig. 4a). In contrast, the CS-SNAP hydrogel groups exhibited significantly fewer colonies on agar plates (Fig. 4a), achieving a killing ratio of over 99% (Fig. 4d and e). The statistical difference between the CS and CS-SNAP groups highlighted the specific quantitative contribution of NO release, which eliminated the residual bacterial population that survived from chitosan contact, thereby bridged the gap between bacterial inhibition and eradication. These results from agar plating assays suggest that the sustained release of NO acts synergistically with chitosan to enhance bactericidal efficacy.

Moreover, clinically, *Pseudomonas aeruginosa* (*P. aeruginosa*) establishes bacterial biofilms, which act as antimicrobial mechanical barriers [48]. This mechanism prevents wound healing efficacy in diabetic patients [49]. Herein, the anti-biofilm activity of the hydrogels was evaluated using of *P. aeruginosa*. The control (medical gauze) or CS-SNAP hydrogels were co-cultured with *P. aeruginosa* for 5 days at 37 °C in darkness. Subsequent crystal violet staining demonstrated that minimal biofilm was formed in the CS-SNAP hydrogel groups compared to the control (Fig. 4c, Fig. S9), and all CS-SNAP hydrogel groups effectively inhibited biofilm formation (Fig. 4f). Collectively, the bacterial co-culture, agar plate assay, and biofilm assay confirmed the potent synergistic antibacterial capability of the CS-SNAP hydrogels. Based on the *in vitro* results regarding cell proliferation, migration, angiogenesis, and antimicrobial activity, we selected CS-SNAP_0.5_ hydrogel for the *in vivo* experiment.

### 2.5 Wound healing of CS-SNAP hydrogels in diabetic rats

The stages of wound healing generally proceed in four steps, including hemostasis, inflammation, proliferation and maturation [2,7]. Based on the above *in vitro* evaluation, we assumed that CS-SNAP hydrogels could accelerate the diabetic wound healing through the promotion of cell proliferation and migration, anti-inflammation and cell regulation functions.

**Fig. 5:**
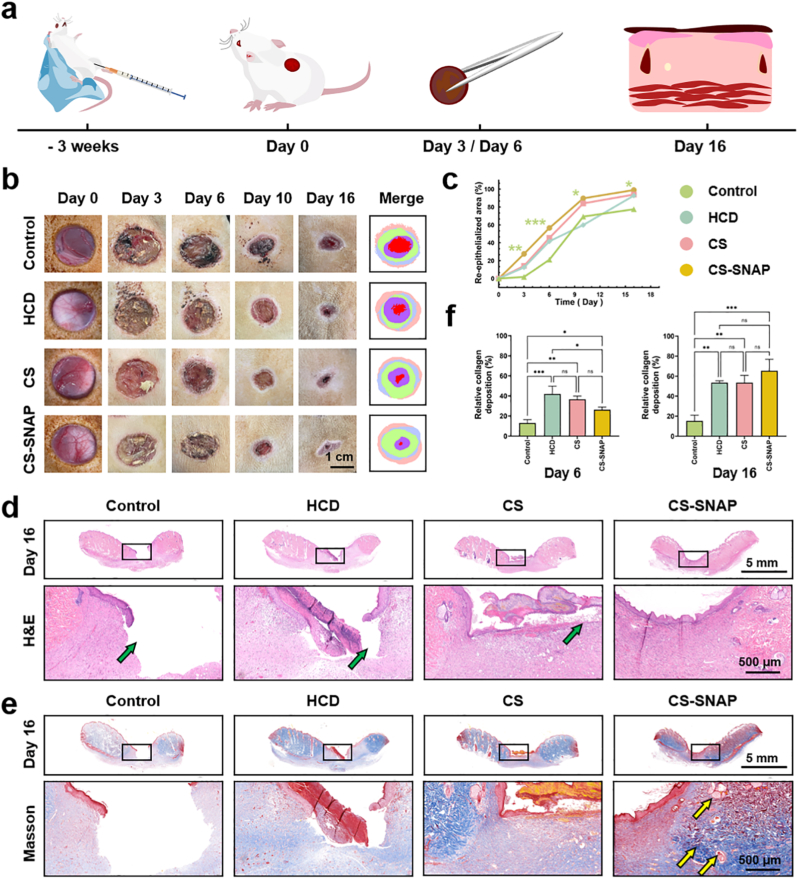
Evaluation of wound healing efficacy in diabetic rats. (a) Scheme of wound healing experiment on full-thickness skin wound model in diabetic rats. (b) Representative images of skin wounds treated with different groups on different days. The wounds were treated with Control, HCD, CS and CS-SNAP_0.5_ hydrogels. (c) Quantitative analysis of re-epithelialized area at the wound sites during healing process. (d) Representative H&E and (e) Masson's staining images of wound tissues from different groups on day 16 post-wounding. Green arrows represent tissue defects; yellow arrows represent wound repair structures. (f) Quantitative analysis of collagen deposition in wound tissues from different groups on day 6 and 16 post-wounding. ∗*p* < 0.05, ∗∗*p* < 0.01, ∗∗∗*p* < 0.001, and ns means not significant (*p* > 0.05).

To investigate the efficacy of CS-SNAP hydrogel in promoting diabetic wound healing, a full-thickness wound with a diameter of 18 mm was created on the dorsal skin of diabetic rats (Fig. 5a). Then, CS hydrogels and CS-SNAP hydrogels were applied to the full-thickness diabetic wounds, and the healing process was monitored during the following 16 days (Fig. 5a). Medical gauze served as the negative control, while commercialized Comfeel® Hydrocolloid dressing (HCD) was used as positive control. The wound size changes of diabetic rats on days 0, 3, 6, 10, and 16 post-surgeries were analyzed. Gross observations of wound closure indicated that diabetic wounds in all treatment groups gradually decreased over time, with a significant decrease in wound size on day 10 and day 16 (Fig. 5b). The negative control group showed a slower rate of wound reduction over the experimental period (Fig. 5b). In contrast, CS-SNAP hydrogel exhibited the most effective pro-healing ability, with the greatest wound closure observed by day 16 (Fig. 5b). Consistent with this observation, quantitative analysis of wound closure rate revealed that the CS-SNAP hydrogel group exhibited a faster healing rate throughout the healing process, achieving a wound closure rate of 89.8% by day 10 and of 99.0% by day 16, respectively (Fig. 5c). The final closure rates by day 16 increased to 92.8% for HCD group, 93.9% for CS hydrogel group, and only 77.6% for the control group (Fig. 5c). These results showed that the CS-SNAP hydrogel achieved the best acceleration effect on diabetic wound healing.

To further understand the pathological healing of wounds with different treatment groups, H&E and Masson's staining of wound tissues were performed on day 6 and day 16 post-wounding. On day 6 post-wounding, the CS-SNAP hydrogel group showed minimal signs of inflammation (Fig. S10). All treatment groups exhibited marked granulation tissue formation except for the control group (Figure S10a, Figure S11a). Granulation tissue thickness in the CS-SNAP hydrogel group measured at 1657 μm ± 156 μm on day 6, significantly higher than that in the control groups (Fig. S11a). Moreover, CS-SNAP hydrogel treatment group showed more evidence of capillaries compared to the control group (Fig. S10b, purple arrows).

By day 16 post-wounding, tissue defects existed in the negative control and the positive control of HCD group (Fig. 5d and e, green arrows), probably due to the tissue fragility. The CS hydrogel group failed to achieve complete re-epithelialization (Fig. 5d and e). In contrast, the CS-SNAP hydrogel group developed a continuous epithelial layer, exhibited the shortest wound length, and presented the most blood vessels, adnexa, and other wound repair structures (Fig. 5e, yellow arrows). On Day 16, there were no significant differences in granulation tissue thickness measurements among all treatment groups, while measurements could not be taken in the control group due to severe tissue defects (Fig. S11b).

Quantitative analysis of collagen deposition was evaluated on day 6 and day 16 post-wounding. On day 6, collagen deposition in the CS-SNAP hydrogel group was not the highest, which reached only 26.3% ± 2.6% (Figure S10b, Fig. 5f). However, on day 16, the collagen deposition rate of the CS-SNAP hydrogel group was the highest among four treatment groups, achieved as high as 65.4% ± 11.5% (Fig. 5e and. f).

Meanwhile, the *in vivo* compatibility of hydrogels was evaluated by applying the hydrogel to the diabetic wounds until complete wound healing. On day 16 post-wounding, H&E staining of vital organs (including heart, liver, spleen, lungs, kidneys) showed that the tissue structures from CS-SNAP groups were consistent with those from the control group and appeared normal (Fig. S12), further confirming the safety of the CS-SNAP hydrogel for application in diabetic wound healing.

The above data demonstrated that CS-SNAP hydrogel accelerated the diabetic wound healing, via regulation of the granulation tissue, contraction of the wound, and deposition of collagen components. Moreover, CS-SNAP hydrogel was able to accelerate the healing process of infected diabetic wounds as compared to the control group (Fig. S13).

### 2.6 Immunofluorescence analysis

**Fig. 6:**
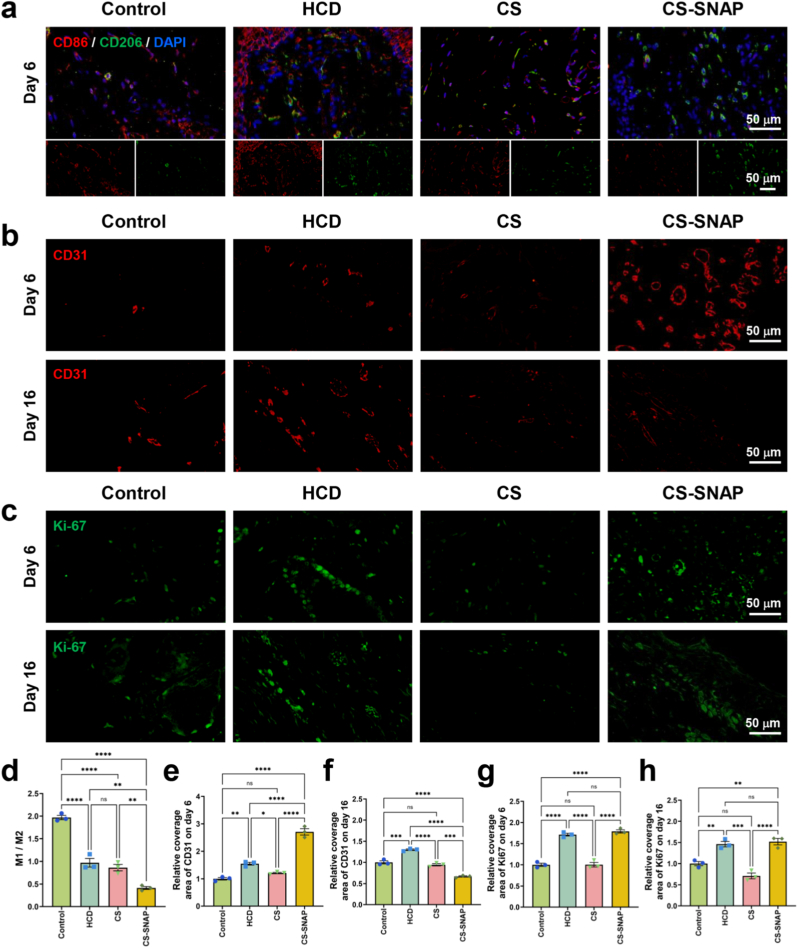
Immunofluorescence (IF) staining analysis of skin tissues post-diabetic wound healing. The wounds were treated with different dressings, including Control, HCD, CS and CS-SNAP_0.5_ hydrogels. (a) IF images of macrophages with CD86 (red), CD206 (green), and nuclei (blue) in skin tissue at wound area on day 6 post-wounding. (b) IF images of angiogenesis by CD31 (red) in skin tissue at wound area on day 6 and day 16 post-wounding, respectively. (c) IF images of proliferation by Ki-67 (green) in skin tissue at wound area on day 6 and day 16 post-wounding, respectively. (d) Ratio of M1 to M2 macrophages in wound tissues, which is calculated by the ratio of CD86^+^ area to CD206^+^ area. (e-f) Quantitative analysis of relative coverage of CD31 in wound skin tissues on day 6 and day 16 post-wounding. (g-h) Quantitative analysis of relative intensity of Ki-67 in wound skin tissues on day 6 and day 16 post-wounding. ∗*p* < 0.05, ∗∗*p* < 0.01, ∗∗∗*p* < 0.001, ∗∗∗∗*p* < 0.0001 and ns means not significant (*p* > 0.05).

Diabetic wounds are chronic and characterized by persistent inflammation [50]. As known, M1 macrophages are responsible for secreting pro-inflammatory cytokines and chemokines, and presenting antigens, thereby initiating immune responses and playing a role in immune surveillance [51]. In contrast, M2 macrophages secrete anti-inflammatory cytokines such as Arginase-I, IL-10, and TGF-β, which reduce inflammation and suppress immune activity, playing a crucial role in wound healing and tissue repair [51]. Therefore, accelerating the polarization of macrophages from the M1 to M2 phenotype is key to resolving chronic inflammation. As previously shown in the H&E staining of the wound tissues on day 6 (Fig. S8a), all experimental groups exhibited signs of inflammation, except the CS-SNAP hydrogel group. Here, immunofluorescence (IF) staining of CD86 and CD206 was further performed for analysis, which are main markers of M1 and M2 macrophages, respectively. Compared to the control group, a downward trend in the M1/M2 ratio was revealed in three treatment groups by day 6 (Fig. 6a and. d), indicating macrophage polarization towards the anti-inflammatory M2 phenotype. This polarization effect from M1 to M2 macrophage was most pronounced in the CS-SNAP hydrogel group, as shown by the most significant green fluorescence of CD206 (Fig. 6a), and the lowest value of M1/M2 ratio (Fig. 6d).

Angiogenesis is another critical factor in diabetic wound healing, as new blood vessels provide oxygen and nutrients to the surrounding cells [52]. Previous *in vitro* results demonstrated that CS-SNAP hydrogel had a strong pro-angiogenic effect (Fig. 3e and f). However, its influence on angiogenesis in diabetic wounds required further clarification. To assess neovascularization, dual immunofluorescence staining with CD31 and Ki-67 was performed, which are specific markers for vascularization and cell proliferation, respectively [53,54]. On day 6, the CS-SNAP hydrogel group exhibited significantly higher expression of CD31 and Ki-67 as compared to the other groups (Fig. 6b–c, Fig. 6e and. g), suggesting the most significant angiogenic activity and higher proliferation in CS-SNAP hydrogel group. By day 16, CD31 expression in the CS-SNAP group decreased compared to the other groups (Fig. 6b and. f), while Ki-67 levels in the CS-SNAP group remained relatively high levels (Fig. 6c and. h). This may be attributed to the faster wound healing in the CS-SNAP group, where granulation tissue had reduced in the later stages (Fig. S11), leading to a decline in CD31 expression by day 16. However, cell proliferation was still ongoing, as evidenced by the sustained Ki-67 levels (Fig. 6c and. 6g–h). These results suggest that CS-SNAP hydrogel not only promoted macrophage polarization to accelerate the anti-inflammatory process in chronic diabetic wounds, but also regulated angiogenesis and stimulated cell proliferation, contributing to more efficient wound healing.

## 3 Discussion

Chronic diabetic wounds pose a significant clinical burden due to their recalcitrant nature, characterized by a hyperglycemic microenvironment, persistent inflammation, and impaired vascularization [7]. A critical underlying pathology is the deficiency of endogenous NO, a pivotal gasotransmitter that regulates inflammation and angiogenesis [8,9]. In this study, we successfully engineered an environmentally activatable CS-SNAP hydrogel platform by grafting SNAP onto the matrix of carboxymethyl chitosan methacryloyl (CMCSMA), and by subsequent fast photopolymerization (Fig. 1a). This platform enables spatiotemporally programmed NO delivery under ambient light and temperature, effectively orchestrating the transition from the inflammatory to the proliferative phase and promoting neovascularization during diabetic wound healing (Fig. 1b and c).

While the therapeutic potential of NO in wound healing is well-established [[16], [17], [18]], the clinical translation of NO-releasing biomaterials has been hampered by the burst release phenomenon and the intrinsic short half-life of NO gas. Previous strategies, such as encapsulating organic nitrates or NONOates into scaffolds, primarily rely on spontaneous hydrolysis [55,56]. Although effective in the short term, these delivery systems often lack precise control over release kinetics. In contrast, our study introduces an advanced environmentally activatable strategy. By covalently grafting the SNAP donor onto the polymer matrix, we engineered a chemically stable system that not only minimizes donor leakage, but also achieves a sustained NO release for over 300 min, triggered strictly by ambient light and physiological temperature. Furthermore, compared to complex multi-step fabrication processes [[16], [17], [18]], our hydrogel features ultrafast *in situ* photopolymerization process (within 10 s) (Figs. 1a and. 2a). This allows for seamless adaptation to irregular diabetic ulcer morphologies, offering a significant clinical advantage over pre-formed dressings.

The rational design of wound dressings is critical to their therapeutic outcomes. Hydrogels are considered ideal candidates for wound management as they can mimic the extracellular matrix (ECM) and maintain a moist healing environment [24,25]. Herein, the interconnected porous structure of the CS-SNAP hydrogels facilitates effective gas exchange and exudate absorption (Fig. 2c). Importantly, its on-demand release mechanism enables the synchronization of NO supply with the dynamic requirements of the wound healing cascade, providing an initial burst release for bacterial clearance, followed by a sustained low-dose release to promote angiogenesis, thereby avoiding the cytotoxicity associated with high-concentration bursts.

Bacterial colonization and biofilm formation are primary drivers for the impaired healing of diabetic wounds in the inflammatory phase. Herein, CS-SNAP hydrogels demonstrated superior antibacterial efficacy, eliminating over 99% of *S. aureus* and *E. coli* (Fig. 4a–b, Fig. 4d and e). This potent activity is attributed to the synergistic mechanism between the positively charged chitosan backbone, which disrupts bacterial membranes, and the sustained release of NO, which induces nitrosative stress in pathogens [34,55]. More importantly, the hydrogel significantly inhibited the formation of *P. aeruginosa* biofilms compared to the control group (Fig. 4c and. f). Given that biofilms serve as mechanical barriers against antibiotics and host immune cells, the capacity of CS-SNAP hydrogel to prevent biofilm establishment offers a crucial advantage in the early management of infected diabetic wounds.

Another critical impediment to diabetic wound healing is the failure of macrophages to transition from the pro-inflammatory M1 phenotype to the reparative M2 phenotype [51]. While the control group exhibited persistent inflammatory responses on day 6, CS-SNAP hydrogel group presented with minimal signs of inflammation (Fig. S10a). Immunofluorescence analysis revealed a significantly decreased M1/M2 ratio in CS-SNAP group, characterized by downregulated CD86 expression and upregulated CD206 expression (Fig. 6a and. d). This suggests that the controlled release of NO functioned as an immunomodulator, actively promoting the resolution of inflammation. By facilitating this phenotypic switch, CS-SNAP hydrogel fostered a microenvironment conducive to the secretion of anti-inflammatory cytokines and growth factors, which are indispensable for the subsequent proliferative phase.

Following the resolution of inflammation, revascularization is also important for supplying oxygen and nutrients to the regenerating tissue [17,52]. *In vitro* results demonstrated that CS-SNAP hydrogel significantly promoted the proliferation and migration of HSF cells and enhanced tube formation of HUVECs (Fig. 3). *In vivo*, this translated to robust angiogenesis, as evidenced by the high expression of CD31 and Ki-67 within the wound bed on day 6 (Fig. 6b–c, Fig. 6e and. g). The observed upregulation of CD31 and Ki-67 was strongly supported by the canonical NO signaling pathway. NO diffused into cells and activated the soluble guanylate cyclase (sGC), leading to elevated intracellular cyclic guanosine monophosphate (cGMP) levels [57]. This secondary messenger activated protein kinase G (PKG), which is known to upregulate the expression of vascular endothelial growth factor (VEGF) [58]. As a potent mitogen, VEGF directly stimulated endothelial cell proliferation (evidenced by high Ki-67 expression) and migration to form new vascular networks (evidenced by increased CD31 coverage). Furthermore, NO has been reported to stabilize hypoxia-inducible factor 1-α (HIF-1α) by inhibiting prolyl hydroxylases, further amplifying angiogenic signaling even in the complex diabetic microenvironment [59,60]. Interestingly, by day 16, CD31 expression decreased in CS-SNAP hydrogel group, while Ki-67 levels remained high (Fig. 6b–c, Fig. 6f and 6h). This pattern suggests a physiological maturation of the vascular network, where rapid initial vessel formation was followed by a stabilization phase as the wound healed.

A critical consideration raised regarding this therapy is whether the duration of NO release (approximately 300 min) is sufficient to support the prolonged diabetic wound healing process. This environmentally activatable NO release was proposed to function as a molecular trigger, which initiated a self-sustaining therapeutic cascade that persisted long after the NO donor was depleted. First, in terms of signal transduction, NO release acted as an immediate signaling molecule that activated the above described sGC-cGMP pathway and stabilized HIF-1α. Once these intracellular signaling switches were activated and downstream gene expression programs were initiated, the physiological effects can persist for days in the absence of extracellular NO. Second, regarding immunomodulation, the initial hours of NO release represented a decisive window for macrophage plasticity. The NO release during several hours effectively promoted the polarization of macrophages towards the pro-reparative M2 phenotype. These reprogrammed macrophages subsequently drove the wound healing process by secreting endogenous anti-inflammatory cytokines and growth factors, thereby maintaining a regenerative microenvironment. Third, for antimicrobial efficacy, the high local concentration of NO during the initial hours targeted the bacteria during their vulnerable lag phase, effectively disrupted their adhesion, and prevented the establishment of recalcitrant biofilms. Finally, this temporal restriction of NO release offered a distinct safety advantage. While continuous high-concentration NO exposure posed risks of nitrosative stress and cytotoxicity in healthy tissues, our strategy employed a controlled therapeutic NO release to maximize efficacy while minimized side effects. This safety profile was confirmed by the excellent biocompatibility and lack of systemic toxicity observed in our studies (Fig. 3, Fig. S12).

The synergistic integration of antibacterial activity, immunomodulation, and angiogenesis significantly accelerated the diabetic wound healing process. Specifically, CS-SNAP hydrogel achieved a 99.0% wound closure rate by day 16, outperforming both CS hydrogel and HCD groups (Fig. 5b and c). Histological analysis further confirmed the superior quality of healing (Fig. 5d and e). CS-SNAP hydrogel group exhibited the thickest granulation tissue on day 6 (Fig. S11a) and the highest collagen deposition on day 16 (Fig. 5f). Furthermore, the regeneration of skin appendages and the formation of a continuous epithelial layer (Fig. 5e) suggest that this therapeutic strategy not only accelerated wound closure but also promoted functional skin regeneration.

Despite these promising results, certain limitations warrant further investigation. First, the rat model relies heavily on wound contraction for closure [61], whereas human wound healing is primarily driven by re-epithelialization. Future studies using porcine models may better predict clinical outcomes. Second, while the light/heat-triggered release mechanism is effective for superficial wounds, the penetration depth of UV/visible light is inherently limited. Developing NO donors responsive to near-infrared (NIR) light could expand the therapeutic application of this technology to deeper tissue injuries. Additionally, NO release kinetics *in vivo* may deviate from the *in vitro* profiles due to the complex physiological environment, such as the absorption of wound exudates and dynamic changes in hydrogel thickness. However, the superior wound healing outcomes, strongly suggest that a therapeutically effective dose of NO was successfully delivered to the target tissue despite these environmental variables. In conclusion, the CS-SNAP hydrogel represents a versatile, environmentally responsive therapeutic platform that successfully corrects the pathological microenvironment of diabetic wounds, offering a potent strategy for clinical chronic wound management.

## 4 Conclusion

In conclusion, we successfully developed an environmentally activatable hydrogel as a proof-of-concept for spatiotemporally programmed NO delivery in diabetic wound healing. This CS-SNAP hydrogel platform successfully translates the external heat and light stimuli into on-demand NO release, thereby orchestrating the complex cascade of diabetic wound repair. This environmentally activatable therapy significantly improved the proliferation, migration, and angiogenic capacity of cells, and showed potent antibacterial activity. In diabetic wound model in rats, CS-SNAP hydrogel promoted neovascularization and cellular proliferation, facilitating rapid granulation tissue formation, re-epithelialization, and collagen remodeling at the wound sites, ultimately accelerated the overall healing process. Furthermore, the CS-SNAP hydrogel treated wounds showed a greater abundance of skin appendages and enhanced skin elasticity, positioning it as a highly promising therapeutic candidate for chronic wound management and skin regeneration.

## 5 Materials and methods

### 5.1 Materials

Carboxymethyl chitosan methacryloyl (CMCSMA), S-Nitroso-N-acetyl-DL-penicillamine (SNAP), N-(3-dimethylaminopropyl)-N′-ethylcarbodiimide (EDC), N-hydroxysuccinimide (NHS), lithium phenyl(2,4,6-trimethylbenzoyl)phosphinate (LAP), were purchased from Shanghai Aladdin Biochemical Technology Co., Ltd. All other chemical reagents are of analytical grade.

### 5.2 Preparation and characterization of hydrogels

#### 5.2.1 Synthesis of CS-SNAP hydrogels

First, a photoinitiator stock solution was prepared by dissolving LAP (0.25% w/v) in deionized (DI) water at 45 °C with shaking for 15 min. Next, CMCSMA was added to the LAP solution to reach a final concentration of 2% (w/v) and stirred in the dark at 45 °C for 2 h to ensure complete dissolution. The solution was then sterilized by heating at 80 °C for 20 min followed by rapid cooling in an ice-water bath. Simultaneously, the SNAP activation solution was prepared. EDC (64.8 mM) and NHS (64.8 mM) were dissolved in DI water, and the pH was adjusted to 5.0. SNAP (54.5 mM) was then added to this solution, and the mixture was maintained at pH 3.0 and stirred in the dark at 4 °C for 2 h to activate the carboxyl groups of SNAP. Subsequently, the activated SNAP solution was mixed with the sterile CMCSMA solution. The pH of the reaction system was adjusted to 4.0 to facilitate the amide coupling reaction, which proceeded in the dark at 4 °C overnight (Fig. 1a). Finally, CS-SNAP hydrogels were obtained by photopolymerization of the mixed solution under ultraviolet (UV) light irradiation (405 nm) for 10 s.

The hydrogels prepared with different SNAP loading ratios (0.25, 0.5 and 1.0, referring to the volume ratio of the SNAP solution to the CMCSMA solution) were labeled as CS-SNAP_0.25_, CS-SNAP_0.5_, and CS-SNAP_1.0_ hydrogels, respectively. The photopolymerization of 2% (w/v) CMCSMA solution without addition of SNAP was labeled as CS hydrogels and served as the control group.

#### 5.2.2 Physicochemical characterization of hydrogels

The condition and appearance of the hydrogels were monitored using a high-resolution cell phone (Apple iPhone 15). The transparency of the prepared hydrogels was evaluated by a UV–Vis spectrophotometer (Cary 5000, Agilent). Transmittance of the hydrogels with a thickness of 0.5 mm was recorded over the spectral range 400 ∼ 700 nm. The microstructure of the hydrogels was investigated using field emission scanning electron microscopy (SEM, Zeiss SIGMA). The pore sizes of the hydrogels were analyzed using ImageJ software (NIH, USA). The elemental composition of the hydrogels was analyzed by X-ray photoelectron spectroscopy (XPS, Thermo Fisher ESCA-LAB 250XI). And the chemical structure of the hydrogels was analyzed by Fourier transform infrared spectroscopy (FTIR, Thermo Fisher NICOLET6700).

### 5.3 Spatiotemporally controlled NO release kinetics

To evaluate the profile of NO release from hydrogels, 10 mg of CS-SNAP hydrogel was immersed in 2 mL of PBS at 4 °C, 25 °C or 37 °C either in dark or in ambient light condition. The samples were then analyzed using Griess reagent kit (G4410-10G; Sigma-Aldrich) as previously reported [11,17,18]. In brief, at different time points, 50 μL of PBS was collected and thoroughly mixed with an equal volume of Griess reagent in a 96-well plate. The plate was then incubated at 37 °C for 10 min in dark. Afterwards, the absorbance at 540 nm was measured using a microplate reader (VICTOR Nivo Multi Microplate Reader, PerkinElmer). The amount of NO released from the hydrogel was determined by comparing the obtained absorbance with that of a standard solution of sodium nitrite (Fig. S6), which was measured using the same method.

### 5.4 *In vitro* biocompatibility and bioactivity assays

#### 5.4.1 Cell culture

Human skin fibroblast cells (HSF cells, Hunan Fenghui Biotechnology Co., Ltd) were cultured in DMEM medium supplemented with 10% fetal bovine serum (FBS) and 1% penicillin-streptomycin (PS), and incubated in 5% CO_2_ at 37 °C. Similarly, human umbilical vein endothelial cells (HUVEC cells, Hunan Fenghui Biotechnology Co., Ltd) were cultured in 1640 medium supplemented with 10% FBS and 1% PS. Routine amplification of the cells was carried out to ensure their suitability for further experiments including bioactivity and cytocompatibility assays.

#### 5.4.2 Cytocompatibility test

First, CS-SNAP hydrogels with different SNAP ratios were sterilized by 3 rounds of washing in 75% ethanol and PBS at 4 °C in dark. 100 mg of CS-SNAP hydrogel was then incubated with 10 mL of complete cell culture medium at 37 °C with agitation at 100 rpm for 24 h. Afterwards, the supernatant was collected and filtered with a 0.22 μm filter (Yeasen Biotechnology) to produce the hydrogel extract solution.

Cytotoxicity of hydrogels was investigated using CCK-8 assay on HSF cells using the obtained hydrogel extract solution. In brief, HSF cells were seeded at a density of 2000 cells/well in 96-well plate, and adhered to the plate for 24 h, then the culture medium was replaced with 100 μL of hydrogel extract solution. The cell culture medium served as a control medium. The medium was refreshed every 24 h. After 24 h or 72 h incubation, viability of HSF cells was evaluated using CCK-8 kit (Beyotime Biotechnology). In brief, 10 μL of CCK-8 solution was added to each well and incubated for 2 h. The absorbance was recorded at 450 nm using the microplate reader. The relative cell viability was expressed as:

$$Cell\;viability\;\left(\%\right)=\frac{A_{t}\;‐\;A_{0}}{A_{c}\;‐{\;A}_{0}}\times 100\%$$

where *A*_*t*_, *A*_*c*_ and *A*_*0*_ represent the absorbance of the test sample, the control group and the blank group, respectively.

Live/dead staining of HSFs was also performed to assess cell viability. Briefly, HSF cells were seeded in a 6-well plate at a density of 8 × 10^4^ cells/well and adhered for 12 h. Then the cells were cultured with the above hydrogel extract solution. Cells cultured with cell culture medium served as the control group. After further incubation for 48 h, the cells were stained with Calcein AM and propidium iodide (Beyotime Biotechnology), which specifically stained the viable and the dead cells, respectively. The cells were then observed under an inverted fluorescence microscope (IX73, Olympus), to examine the green fluorescence of viable cells and the red fluorescence of dead cells, respectively. All experiments were conducted in triplicate to ensure reproducibility.

#### 5.4.3 Hemocompatibility test

First, the hydrogel extract solution was obtained similarly as above but with saline (0.01M, pH 7.4). Fresh rat citrated blood was diluted with saline (saline: blood = 5: 4). Then, 100 μL of the diluted blood was added to 3 mL of the hydrogel extract solution and incubated for 1 h at 37 °C. Saline-treated blood was used as negative control, and Triton-X-treated blood were used as positive control. Following 5 min centrifugation at 1000 rpm, 100 μL supernatant was added into 96-well plate and the absorbance was measured at 540 nm using a microplate reader. The following formula was used to determine the hemolysis (%):

$$Hemolysis\;\left(\%\right)=\frac{A_{t}\;‐\;A_{n}}{A_{p}\;‐\;A_{n}}\times 100\%$$

where *A*_*t*_, *A*_*p*_ and *A*_*n*_ represent the absorbance of the test sample, the positive control and the negative control, respectively.

#### 5.4.4 Evaluation of cell migration

Migration of HSF cells was investigated using scratch assay. In brief, HSF cells at logarithmic growth stage were seeded at a density of 2 × 10^5^ cells/well in 6-well plate and incubated for 24 h. The monolayer of cells was then scraped with a 200 μL pipette tip, rinsed, and imaged with an inverted microscope (IX73, Olympus). Next, the medium was replaced with the hydrogel extract solution. HSF cells incubated with cell culture medium served as control. The cells were incubated for another 24 h and 48 h, then optical images of cells were obtained using the inverted microscope. The distances between two sides of the scratch were measured using ImageJ software (NIH, Maryland, USA).

#### 5.4.5 Tube formation assay

Tube formation assay was performed to evaluate the angiogenesis potential of hydrogels. In brief, 50 μL of Matrigel (Corning) was added to 96-well plate, followed by gelation at 37 °C for 1 h. Then, HUVEC cells were seeded onto the Matrigel at a density of 2 × 10^4^ cells/well, and incubated in 50 μL of the fresh hydrogel extract solution. HUVEC cells incubated in normal cell culture medium served as control. After 6 h incubation, the formation of capillary-like structures of HUVEC cells was imaged with the inverted microscope. Quantitative analysis of the tubular networks was conducted using angiogenesis analyzer in ImageJ software, including the number of junction points and the total segment length.

### 5.5 *In vitro* antibacterial and anti-biofilm test

First, antibacterial activity of the CS-SNAP hydrogels was evaluated using of *S. aureus* and *E. coli*. In brief, 10 mL suspensions of *S. aureus* or *E. coli* at a concentration of 10^6^ CFU/mL were co-incubated with 50 mg CS-SNAP_0.25/0.5/1.0_ hydrogels in a Luria-Bertani (LB) broth in different tubes at 37 °C overnight in the dark with shaking (100 rpm). Meanwhile, the bacteria co-cultured with medical gauze in LB broth served as control. Next, 100 μL of the bacterial suspension from different groups was smeared on the bacterial culture dish, and the growth patterns of bacteria was observed after 24 h. Meanwhile, 100 μL of the bacterial suspension from different groups was transferred to 96-well plate, and the viability of bacteria was evaluated by CCK-8 assay as above described according to previous report [62]. The killing ratio of bacteria was calculated using the following equation:

$$Killing\;ratio\;\left(\%\right)=\frac{A_{c\;}‐\;A_{t}}{A_{c}}\times 100\%$$

where *A*_*t*_ and *A*_*c*_ represent the absorbance of the test sample and the control group, respectively.

Moreover, the antibacterial activity of biofilm formation of the hydrogels was evaluated using *P. aeruginosa*. Briefly, 3 mL suspensions of *P. aeruginosa* at a concentration of 10^6^ CFU/mL and 50 mg different samples were co-incubated in LB broth in 6-well plate at 37 °C in the dark for 5 days. Afterwards, the bacterial biofilm was fixed with methanol, rinsed, and stained with 1% crystal violet. The stained bacterial biofilm was then dissolved in 33% acetic acid. Then, 100 μL of the suspension was transferred into 96-well plate, and the absorbance was measured at 570 nm using a microplate reader.

### 5.6 *In vivo* diabetic wound healing evaluation

#### 5.6.1 Ethical approval

All animal study was approved by the Institutional Animal Care and Use Committee of Wuhan University (IACUC Number: ZN2024001). Male SD rats (200 ∼ 250 g) were obtained from Wuhan University Center for Animal Experiment.

#### 5.6.2 Establishment of diabetic wound model

Male SD rats were acclimatized for 5 days. After an 8-h fast, rats were intraperitoneally injected with streptozotocin (STZ) at a dose of 75 mg/kg, and this procedure was repeated daily for 5 consecutive days to establish type I diabetic model [39,63,64]. Blood glucose levels were continuously monitored every 3 days for 3 weeks. To confirm the validity of the diabetic model, strictly quantitative criteria were applied: only rats with a random blood glucose level consistently higher than 16.6 mM were considered diabetic model and selected for the subsequent experiments. The animals were then randomly divided into different groups for further experiments.

#### 5.6.3 Application of CS-SNAP hydrogels for diabetic wound healing

Animals were anesthetized by sevoflurane inhalation, and the dorsal hair of diabetic rats was shaved or depilated. A circular biopsy punch with a diameter of 18 mm was used to induce full-thickness skin wounds on the dorsum of diabetic rats. Then, diabetic wounds received different treatments, including medical gauze (Control) as a negative control, commercialized Comfeel® hydrocolloid dressing (HCD) as a positive control, CS hydrogel and CS-SNAP_0.5_ hydrogels. For the hydrogel groups, approximately 100 mg of the sample was applied topically to completely cover the wound area, followed by the application of Tegaderm™ transparent film (3M, USA) to secure the dressing. NO release mechanism of the environmentally activatable hydrogel was designed to be patient-friendly and non-intrusive. Therefore, in practical application, the hydrogels were activated by the physiological body temperature of the rats (∼37 °C) and ambient conditions (∼25 °C, day/night light cycle). No additional external intense heating or UV irradiation was applied to the animals to avoid potential thermal damage or UV-induced phototoxicity. The dressings were changed every 3 days, and the healing process of diabetic wounds was observed and photographed every 3 days.

#### 5.6.4 Wound closure rate analysis

Wound healing process of each group was observed and photographed every 3 days. The wound areas were analyzed using ImageJ software. The wound closure rate was calculated according to the following formula, which represents the change in wound size relative to the original wound size.

$$Wound\;closure\;rate\;\left(\%\right)=\frac{S_{0}\;‐\;S_{n}}{S_{0}}\times 100\%$$

where *S*_*0*_ and *S*_*n*_ represent the wound area on day 0 and on day n, respectively.

#### 5.6.5 Application of CS-SNAP hydrogels for infected diabetic wound healing

Full-thickness diabetic wounds were created using above method. Subsequently, 50 μL of bacterial suspension (*P. aeruginosa* at 10^6^ CFU in PBS) was inoculated onto the wound and left to settle for 10 min, then the wound was covered with transparent Tegaderm™ film (3 M, USA). The wounds were left for 24 h to allow the inoculated bacteria to form biofilm. Afterwards, the infected diabetic wounds with bacterial biofilm received different treatment groups, including medical gauze (Control), Comfeel® hydrocolloid dressing (HCD), CS hydrogels and CS-SNAP hydrogels. The healing process of the infected diabetic wounds was photographed every 3 days.

#### 5.6.6 Histological analysis and immunofluorescence staining

Diabetic wound specimens were collected on the 6th and 16th day post treatment. Hematoxylin and eosin (H&E) staining was conducted to evaluate the epidermal regeneration and the formation of granulation tissue within the diabetic wounds. Masson's trichrome staining was carried out to assess collagen deposition in the wound bed.

Furthermore, immunofluorescence (IF) staining of the regenerated skin was conducted to analyze the tissue inflammatory response (CD86 and CD206), angiogenesis (CD31), and cell proliferation (Ki-67). Briefly, tissue sections were incubated with primary antibodies overnight at 4 °C, including goat anti-mouse monoclonal antibody against CD86 (Abcam, 1:100), rabbit anti-mouse polyclonal antibody against CD206 (Proteintech, 1:200), rabbit monoclonal antibody against CD 31 (Abcam, 1:300), and rabbit polyclonal antibody against Ki-67 (Abcam, 1:100), respectively. Afterwards, the secondary antibody corresponding to the primary antibody with different fluorescence were bounded, where CD86 and CD31 were stained in red fluorescence, CD206 and Ki-67 were stained in green fluorescence. Cell nuclei were stained with 4′,6-diamidino-2-phenylindole (DAPI, Abcam) with blue fluorescence. The slices were protected with antifade reagent, and observed with fluorescence microscopy (Aperio Versa 8, Leica). IF images were quantitatively analyzed by ImageJ software.

### 5.7 Statistical analysis

All experimental data were subjected to statistical analysis, and the results were presented as mean value ± standard deviation (S.D.). Statistical differences were determined using one-way ANOVA and Student's t-test. A significant difference was considered when *p* < 0.05, whereas ∗ *p* < 0.05, ∗∗*p* < 0.01, ∗∗∗*p* < 0.001, ∗∗∗∗*p* < 0.0001 and ns means not significant (*p* > 0.05).

## CRediT authorship contribution statement

**Langjie Chai:** Data curation, Formal analysis, Investigation, Methodology, Writing – original draft. **Yiran Shi:** Data curation, Investigation. **Qianqian Li:** Funding acquisition, Methodology. **Yifan Han:** Data curation, Methodology. **Liangcong Hu:** Funding acquisition, Project administration. **Yifeng Lei:** Conceptualization, Formal analysis, Funding acquisition, Supervision, Writing – review & editing. **Liang Guo:** Funding acquisition, Resources, Supervision.

## Declaration of competing interest

The authors declare no conflict of interest.

## Data Availability

Data will be made available on request.
