# Supplementary material for: Environmentally activatable hydrogel for spatiotemporally programmed nitric oxide delivery for diabetic wound healing
Source: Mater Today Bio. 2026 Feb 10;37:102916. doi: 10.1016/j.mtbio.2026.102916 (PMC12925292; doi:10.1016/j.mtbio.2026.102916)
Supplement: Multimedia component 1 [file mmc1.docx]

**Supporting Information**

Environmentally activatable hydrogel for spatiotemporally programmed nitric oxide delivery for diabetic wound healing

Langjie Chai ^a, b, 1^, Yiran Shi ^a, 1^, Qianqian Li ^a, 1^, Yifan Han ^c^,

Liangcong Hu ^a, *^, Yifeng Lei ^c, d, *^, Liang Guo ^a, *^

a Department of Plastic Surgery, Zhongnan Hospital of Wuhan University, Wuhan 430071, China

b Department of Plastic Surgery, The First Affiliated Hospital of Shandong First Medical University & Shandong Provincial Qianfoshan Hospital, Jinan 250013, China

c School of Power and Mechanical Engineering, Wuhan University, Wuhan 430072, China

d Wuhan University Shenzhen Research Institute, Shenzhen 518057, China

1 These authors contributed equally to this work.

* Corresponding authors. E-mails: guolianghbwh@163.com (L. Guo), yifenglei@whu.edu.cn (Y. Lei), huliangcong@whu.edu.cn (L Hu).


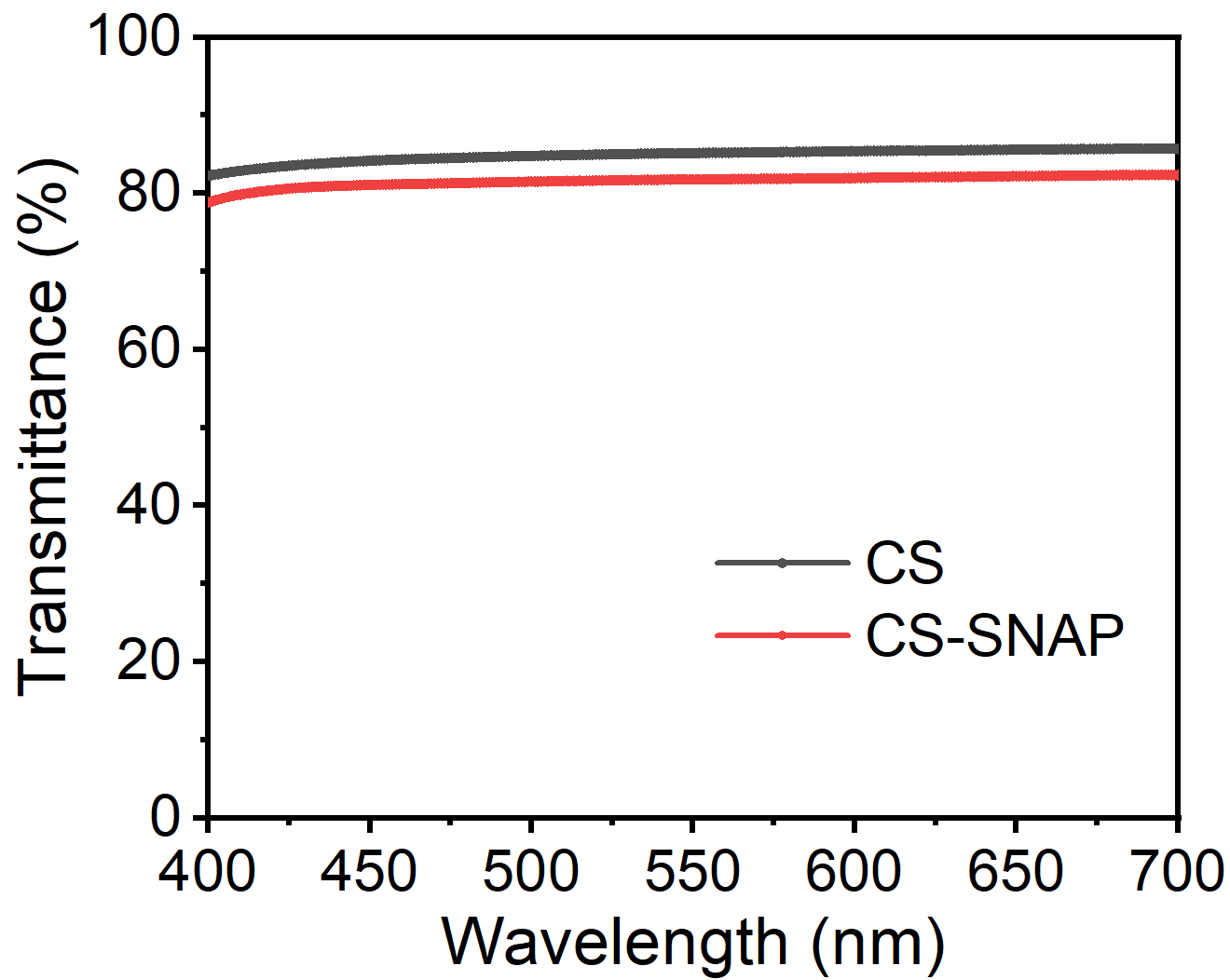


**Figure S1.** Transmittance of CS and CS-SNAP hydrogels in the spectral range of 400 ~ 700 nm.


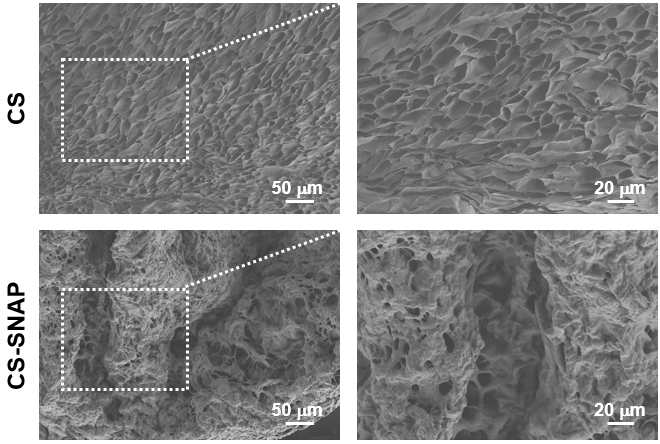


**Figure S2.** SEM images of CS and CS-SNAP hydrogels in lower magnification.


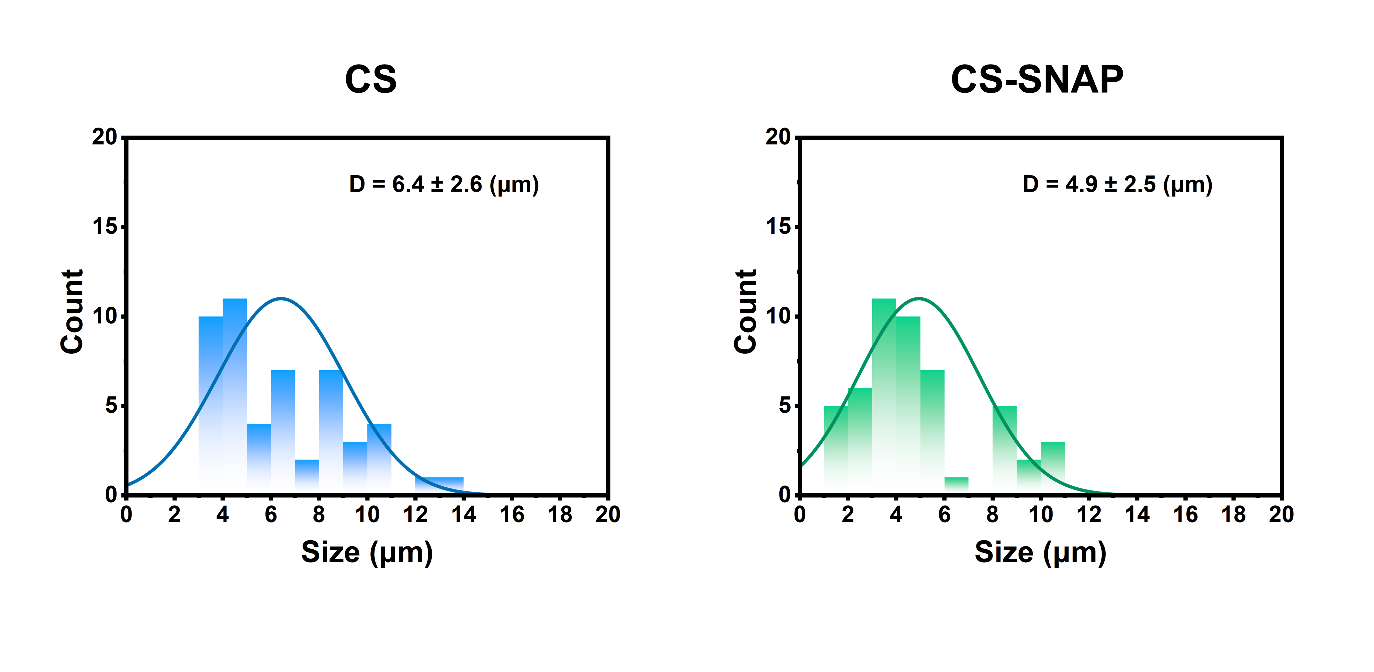


**Figure S3.** Distribution of pore sizes of CS and CS-SNAP hydrogels.


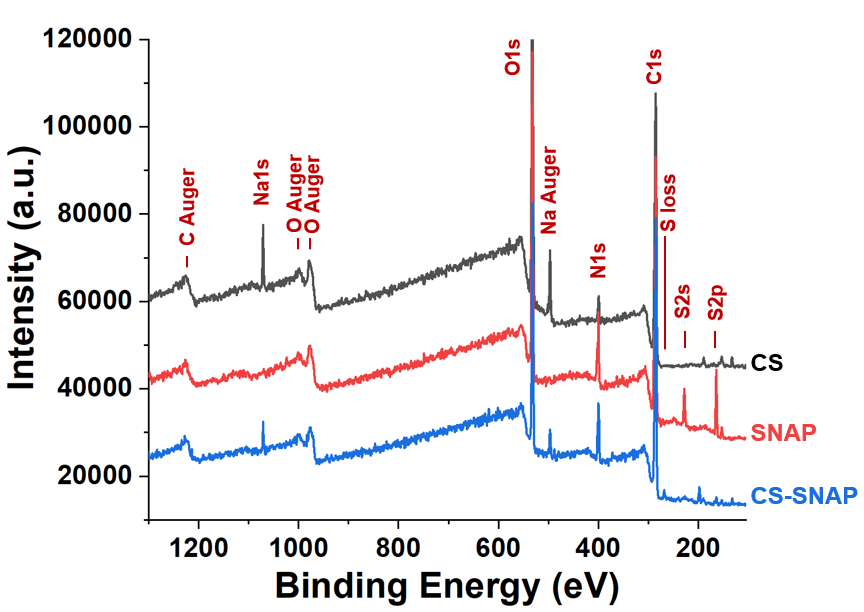


**Figure S4.** XPS full spectra during synthesis of CS-SNAP hydrogels.


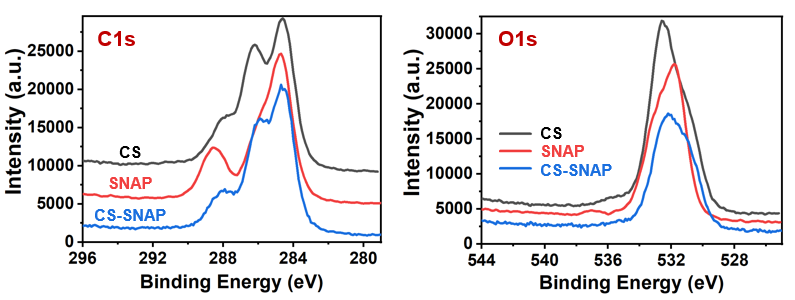


**Figure S5.** XPS high-resolution spectra during synthesis of CS-SNAP hydrogels, including C1s and O1s spectra.


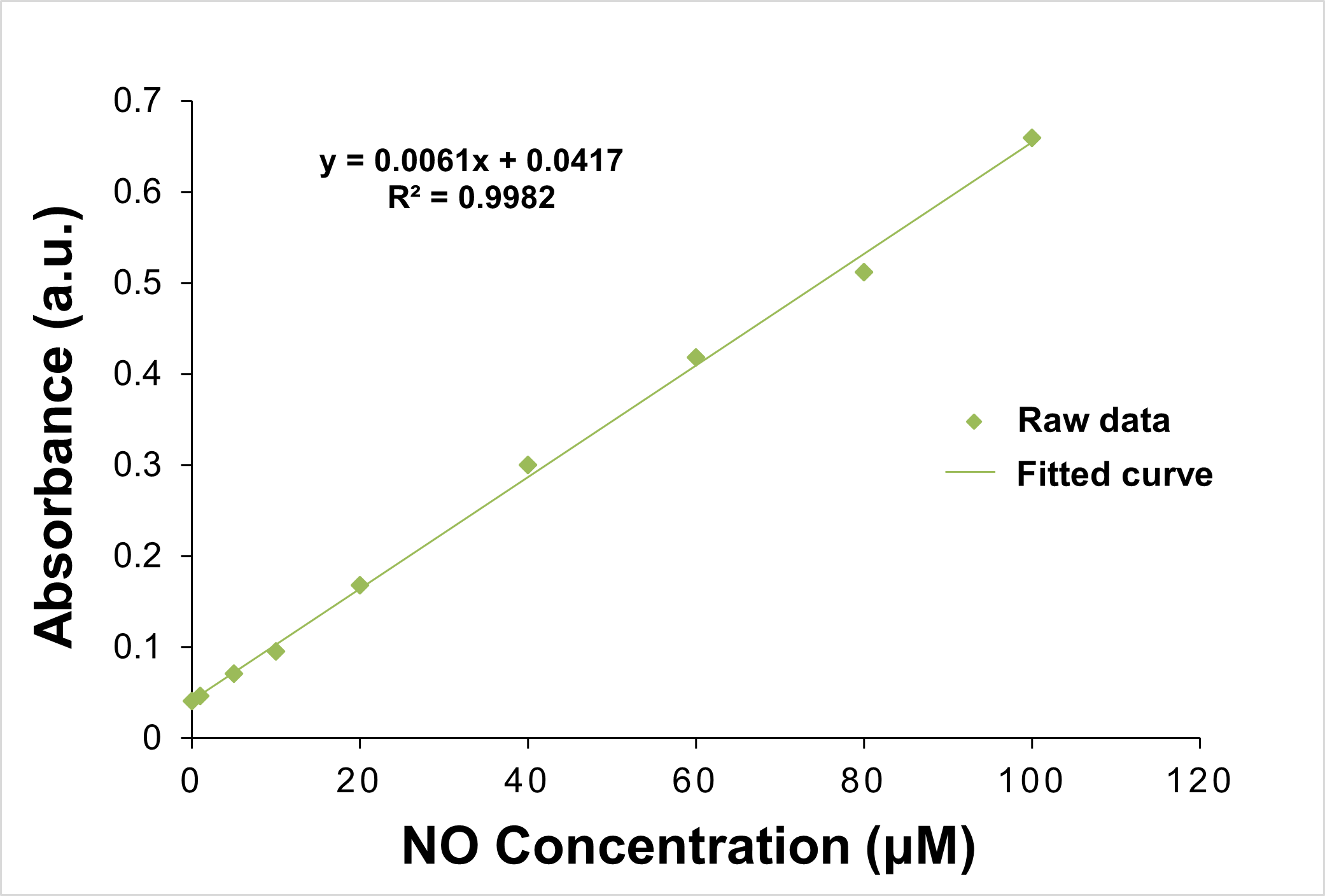


**Figure S6.** Standard curve for NO concentration.


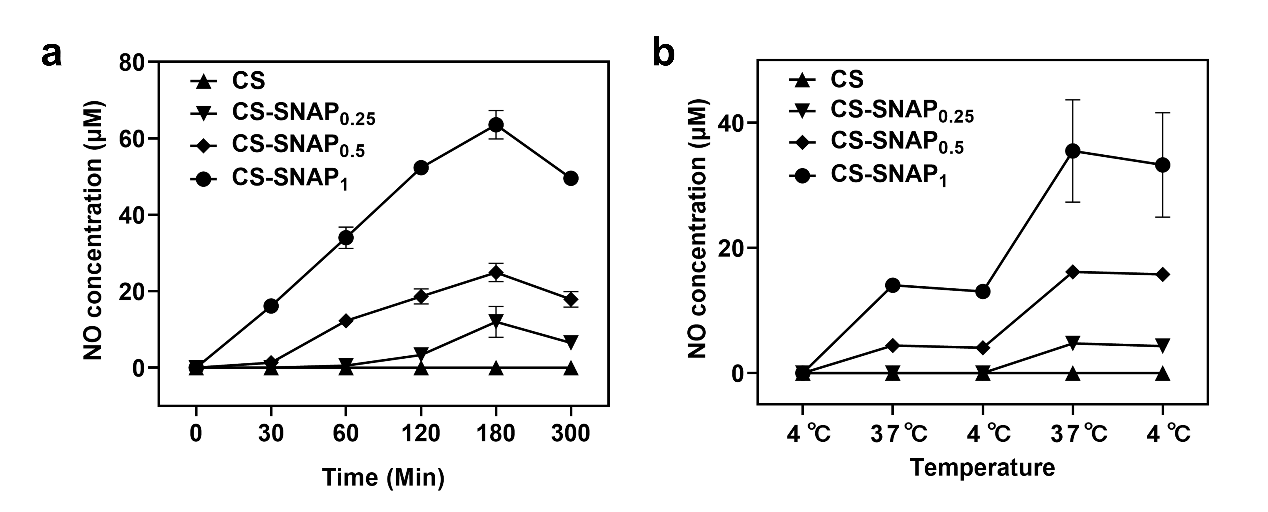


**Figure S7.** (a) Time-dependent release curves of NO from different hydrogel groups (25 °C with ambient light). (b) NO release curves of different hydrogels as a function of temperature (in darkness).


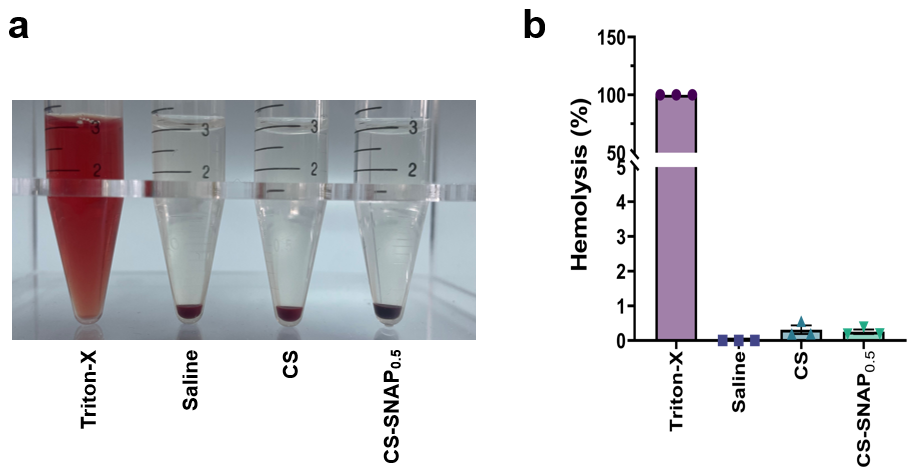


**Figure S8.** Hemolysis test of CS-SNAP hydrogels. (a) Optical image of hemolysis test with different groups. (b) Hemolytic rate of red blood cells treated with different groups.


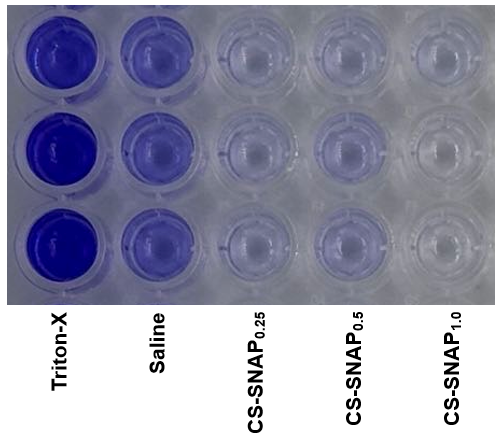


**Figure S9.** Images of dissolved bacterial biofilms of *P. aeruginosa*. treated with different groups.


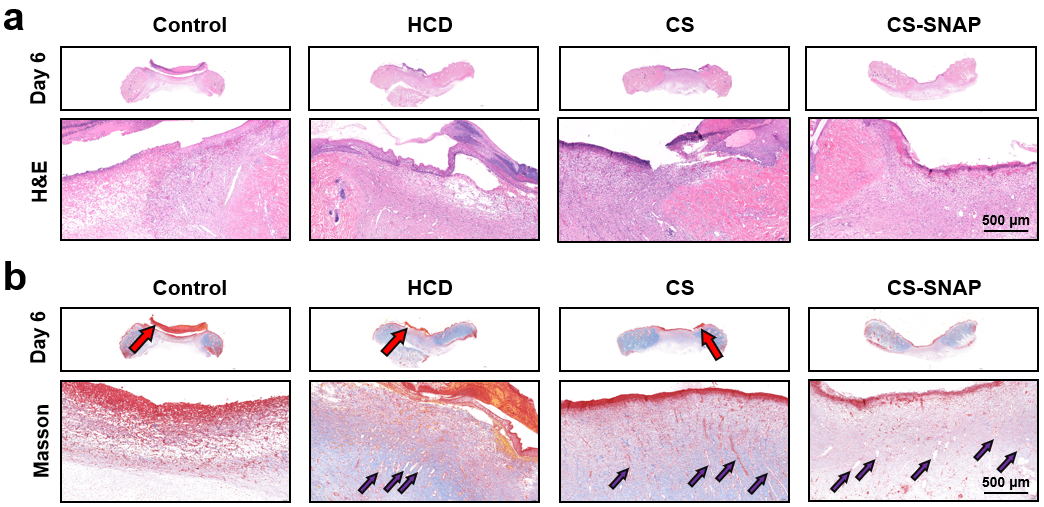


**Figure S10.** (a) Representative H&E and (b) Masson’s staining images of wound tissues from different groups on day 6 post-wounding. Red arrows indicate inflammation, purple arrows indicate new capillary formation.


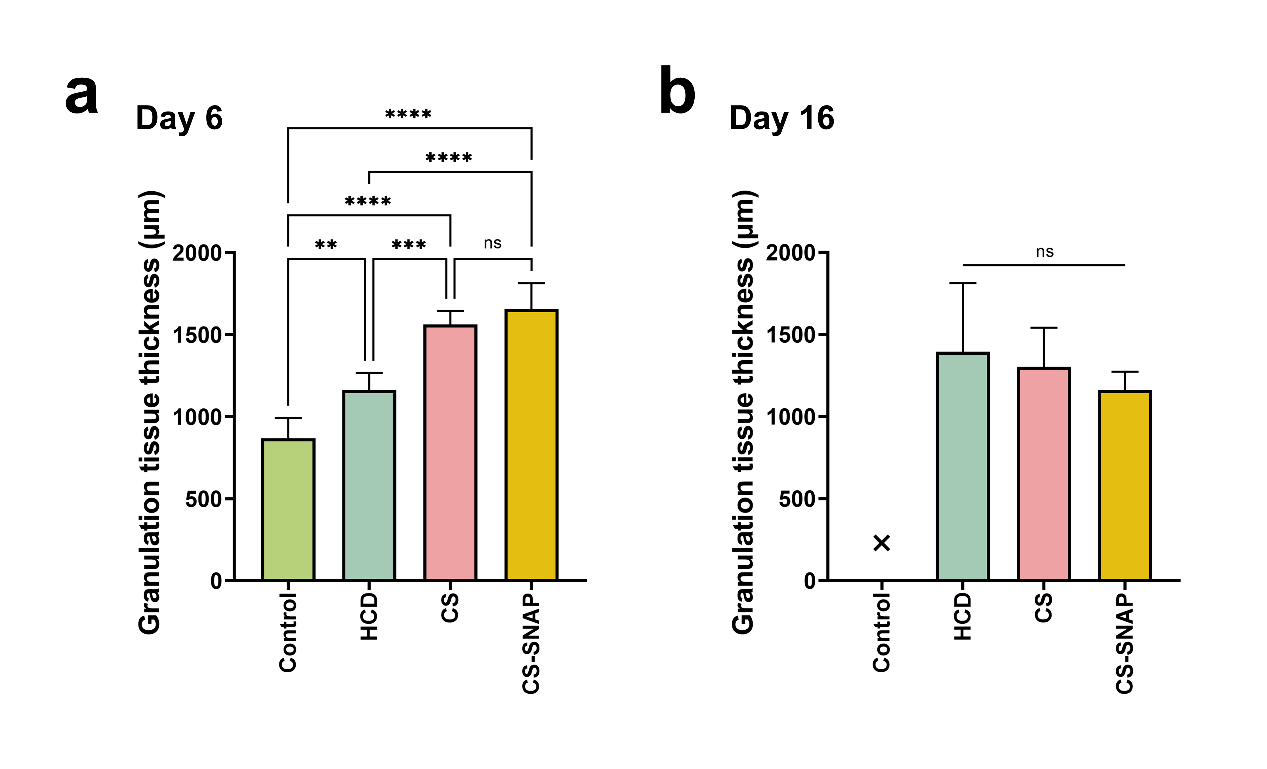


**Figure S11.** Quantitative analysis of granulation tissue thickness in wound tissues from different groups on day 6 post-wounding (a) and on day 16 post-wounding (b).


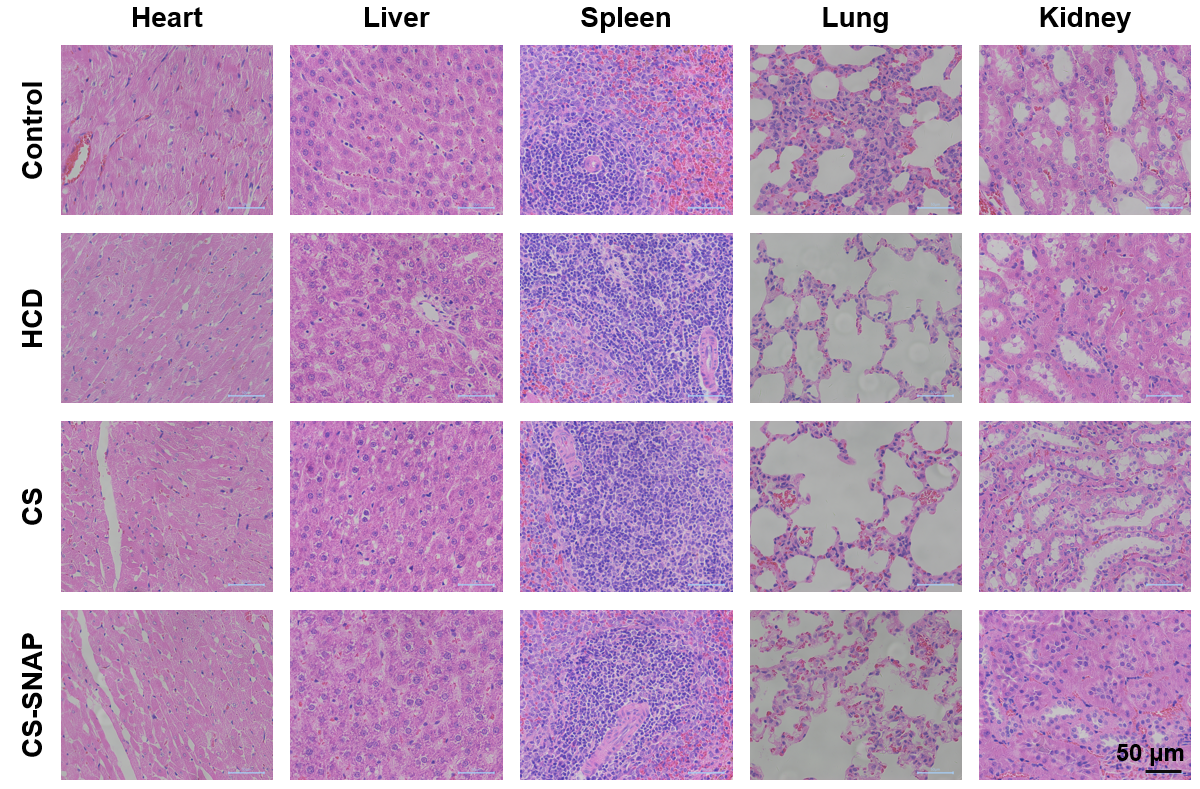


**Figure S12.** H&E staining images of major organs of diabetic rats on day 16 post-wounding.

**Application of CS-SNAP hydrogels for infected diabetic wound healing**

After creation of full-thickness wounds on diabetic rats, *P. aeruginosa* were seeded on the diabetic wounds for 24 h. After the formation of biofilm, the infected diabetic wounds received different treatment groups, including medical gauze (negative control), commercialized Comfeel® hydrocolloid dressing (HCD, positive control), CS hydrogels and CS-SNAP hydrogels.

However, due to the diabetic condition and bacterial infection, most animals eventually died after infection. Among the survival animals, the infected diabetic wounds treated with CS-SNAP hydrogels healed faster than the control group (Figure S13).


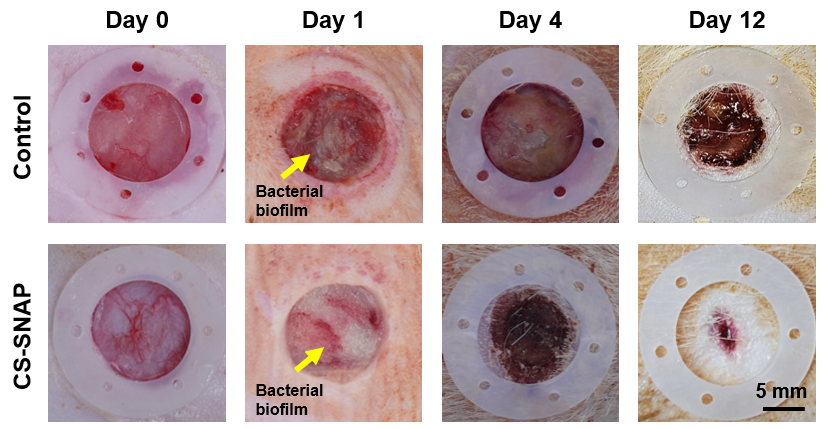


**Figure S13.** Representative images of healing process of *P. aeruginosa* infected skin wounds. The wounds were treated with control group (medical gauze) and CS-SNAP hydrogels from day 1 to day 14.

**Table S1.** Atomic ratio of different samples by XPS analysis.

|  | C1s (%) | N1s (%) | O1s (%) | S2p (%) |
| --- | --- | --- | --- | --- |
| CS | 63.48 | 4.04 | 30.29 | 0 |
| SNAP | 57.76 | 9.41 | 24.88 | 5.74 |
| CS-SNAP | 66.28 | 8.52 | 21.68 | 1.07 |
